# Plasma sphingolipids mediate the association between gut microbiome composition and type 2 diabetes risk in the HELIUS cohort: a case-cohort study

**DOI:** 10.1136/bmjdrc-2024-004180

**Published:** 2024-07-18

**Authors:** Martin F Overbeek, Femke Rutters, Max Nieuwdorp, Mark Davids, Irene van Valkengoed, Henrike Galenkamp, Bert-Jan van den Born, Joline W J Beulens, Mirthe Muilwijk

**Affiliations:** 1 Epidemiology and Data Science, Amsterdam UMC Locatie VUmc, Amsterdam, The Netherlands; 2 Department of Epidemiology and Data Science, Amsterdam Public Health Research Institute, Amsterdam UMC Location AMC, Amsterdam, The Netherlands; 3 Vascular Medicine, Amsterdam UMC Locatie AMC, Amsterdam, The Netherlands; 4 Internal Medicine, Amsterdam UMC Locatie AMC, Amsterdam, The Netherlands; 5 Public and Occupational Health, Amsterdam UMC, Amsterdam, The Netherlands; 6 Public Health, Amsterdam UMC Locatie AMC, Amsterdam, The Netherlands; 7 Epidemiology & Data Science, Amsterdam UMC Locatie VUMC, Amsterdam, The Netherlands; 8 Epidemiology and Data Science, Amsterdam UMC, Amsterdam, The Netherlands

**Keywords:** Type 2 Diabetes, Microbiology, Metabolism

## Abstract

**Introduction:**

The association between the gut microbiome and incident type 2 diabetes (T2D) is potentially partly mediated through sphingolipids, however these possible mediating mechanisms have not been investigated. We examined whether sphingolipids mediate the association between gut microbiome and T2D, using data from the Healthy Life in an Urban Setting study.

**Research design and methods:**

Participants were of Dutch or South-Asian Surinamese ethnicity, aged 18–70 years, and without T2D at baseline. A case-cohort design (subcohort n=176, cases incident T2D n=36) was used. The exposure was measured by 16S rRNA sequencing (gut microbiome) and mediator by targeted metabolomics (sphingolipids). Dimensionality reduction was achieved by principle component analysis and Shannon diversity. Cox regression and procrustes analyses were used to assess the association between gut microbiome and T2D and sphingolipids and T2D, and between gut microbiome and sphingolipids, respectively. Mediation was tested familywise using mediation analysis with permutation testing and Bonferroni correction.

**Results:**

Our study confirmed associations between gut microbiome and T2D and sphingolipids and T2D. Additionally, we showed that the gut microbiome was associated with sphingolipids. The association between gut microbiome and T2D was partly mediated by a sphingolipid principal component, which represents a dominance of ceramide species over more complex sphingolipids (HR 1.17; 95% CI 1.08 to 1.28; proportional explained 48%), and by Shannon diversity (HR 0.97; 95% CI 0.95 to 0.99; proportional explained 24.8%).

**Conclusions:**

These data suggest that sphingolipids mediate the association between microbiome and T2D risk. Future research is needed to confirm observed findings and elucidate causality on a molecular level.

**WHAT IS ALREADY KNOWN ON THIS TOPIC**

**WHAT THIS STUDY ADDS**

- The gut microbiome was associated with circulating sphingolipids, which mediated the association between gut microbiome and incident type 2 diabetes.

**HOW THIS STUDY MIGHT AFFECT RESEARCH, PRACTICE OR POLICY**

- Our results can be taken as a starting point to unravel the exact pathways and involved microbiome species, ultimately interventions may be developed to improve circulating sphingolipid ratios.

## Introduction

 Type 2 diabetes (T2D) is a complex multifactorial disease, with a range of risk factors including age, family history, unhealthy diet and physical inactivity.^1^ Recently, the gut microbiome has been recognized to contribute to obesity, insulin resistance and T2D.^2 3^ The exact mechanisms underlying this association remain unclear^4^ but include synthetization, metabolization and production of metabolites by the gut microbiome, which in turn impact T2D risk. Protective metabolites include short-chain fatty acids and indole derivatives, while branched chain amino acids, trimethylamine-*N*-oxide and imidazole propionate can exert harmful effects.^5 6^ Identification of additional pathways broadens the range of options for clinical practice to prevent and treat T2D.

One group of metabolites with high potential to serve as a pathway through which the gut microbiome affects T2D risk are sphingolipids: bioactive lipids involved in processes ranging from cell proliferation and apoptosis to exosome secretion.^7^ Importantly, they have been identified as risk factors for T2D^8 9^ and mechanisms have been described by which they can impact insulin signaling.^10 11^ Gut microbiota are potentially capable of modifying sphingolipid levels through at least three distinct mechanisms. First, some bacteria, mostly in the phylum *Bacteroidetes*, are known to possess serine palmitoyltransferase (SPT) and are, therefore, capable of synthesizing sphingolipids de novo.^12 13^ Preliminary results indicate that a *Bacteroides* enterotype might convey increased risk of T2D.^14^ Bacterial sphingolipids have been shown to enter human metabolic pathways in vitro,^13^ suggesting that microbiome-derived sphingolipids could have downstream effects in the host. Second, since diet is an important source of sphingolipids, processing ingested sphingolipid species before they are absorbed into the bloodstream can also be a way for microbiota to alter plasma sphingolipid profiles in the host. Finally, microbiota have been found to modulate the intestinal farnesoid X receptor (FXR),^15^ a nuclear receptor with downstream effects on sphingolipid signaling, expressed in intestinal epithelium.

The contribution of the microbiome to concentrations of circulating sphingolipids has not been assessed yet. Additionally, the association of sphingolipids synthesized by the microbiome with T2D risk remains to be investigated. Our objective is, therefore, to (1) confirm the associations between gut microbiome and sphingolipids with incident T2D in our data; (2) investigate whether the gut microbiome is associated with circulating sphingolipids; (3) investigate whether the association between gut microbiome and incident T2D is mediated by sphingolipids in the general population.

## Materials and methods

### Study design

**Figure 1:**
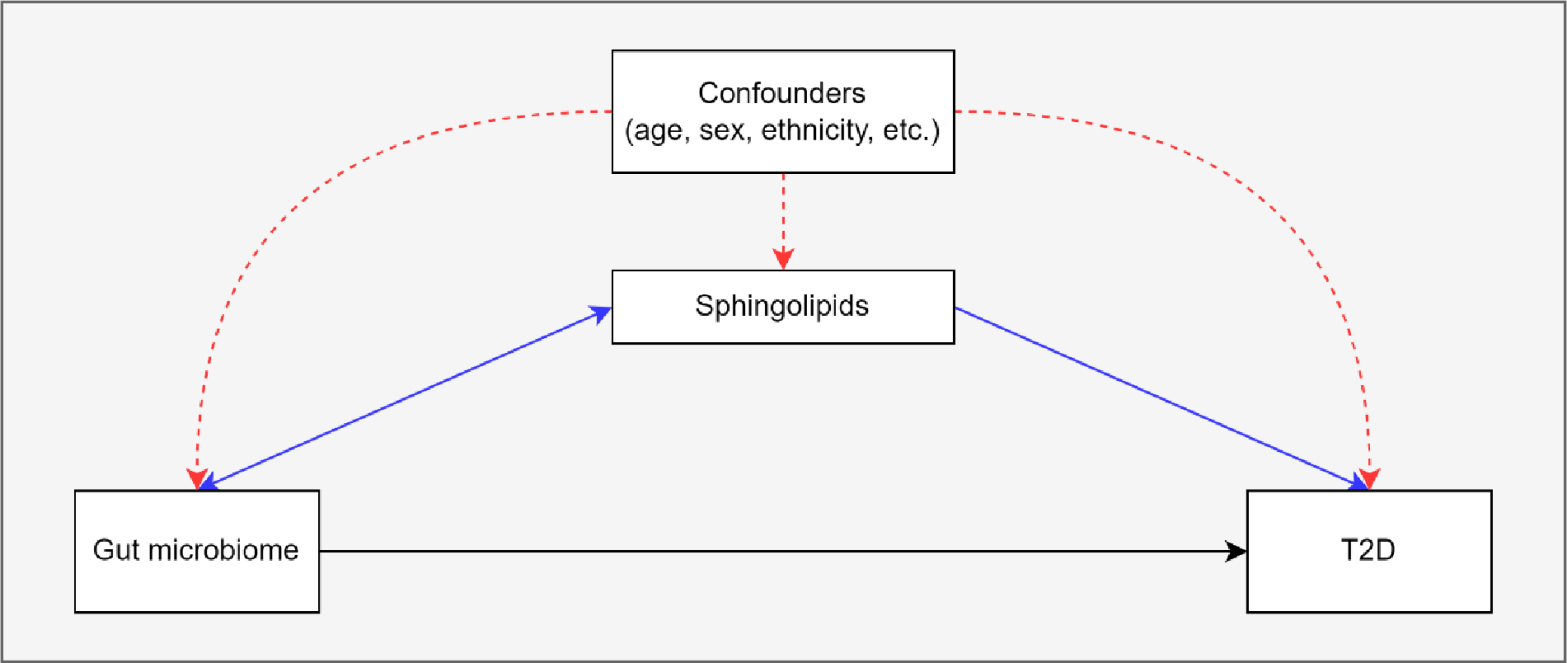
A schematic diagram of the mediation hypothesis. Arrowheads depict direction of effect. The hypothesis is that the association between gut microbiome and incident T2D is partly mediated through host sphingolipid levels (blue arrows). Confounders (red arrows) can affect all variables included in the mediation relationship. T2D, type 2 diabetes.

Figure 1 provides a graphical summary of the hypothesized mediation of the association between gut microbiome composition and T2D by sphingolipids. The hypothesis is assessed in the Healthy Life in an Urban Setting (HELIUS) prospective multiethnic cohort study, in participants of Dutch and South-Asian Surinamese ethnicity.^16^ Inclusion of two ethnic groups increases the variation in gut microbiome composition.^17^ Details of HELIUS, a population-based prospective cohort study, are described elsewhere^16 18^ and are summarized here in brief. Baseline data for the HELIUS study were gathered between 2011 and 2015 and included a physical evaluation, questionnaires and biosample collection (among which blood and feces). Participants were aged between 18 and 70 years and randomly recruited from the Amsterdam municipal registry stratified by ethnicity (n=24 789) (online supplemental file 1). The current study required presence of both blood plasma sphingolipid and gut microbiome data, both of which have been generated in previous HELIUS substudies,^8 19^ which overlapped only partly. Microbiome data were generated in 6032 participants, who handed in a feces sample at baseline. Sphingolipids were determined in a substudy with a case-cohort design, in which participants with and without T2D were compared. The selection of this subcohort was as follows: from the total group of HELIUS participants, those who did not fill out a questionnaire, nor underwent a physical examination were excluded from the study (n=2624). Next, participants with prevalent T2D (n=773), with less than two vials of EDTA plasma available (n=186), without permission for data linkage or storage of biological material (n=671) and of different ethnicity than Dutch or South-Asian Surinamese (14 558) were excluded. This led to 5977 participants who were followed over time, 299 participants were lost to follow-up. From the remaining 5678 participants, 95 participants developed T2D over time and for them sphingolipids were determined. Next to these 95, a total of 700 participants were randomly selected to be included in the subcohort, and sphingolipids were determined, whether they developed T2D or not. Of those participants, 23 participants were lost to follow-up and nine participants developed T2D. The overlap between the two substudies (microbiome and sphingolipids) was 209, of whom 176 in the subcohort (including three cases) and 36 cases. HELIUS was approved by the Institutional Review Board of the Amsterdam UMC (MREC 10/100# 17.10.1729). All participants provided written informed consent before onset of the study.

### Type 2 diabetes

Incident T2D cases were identified through record linkage with two healthcare registrations, as described elsewhere.^8^ In brief, we first linked HELIUS data by Citizen Service number to the Achmea Health Database with registrations from 1 January 2010 until 30 April 2016. Second, HELIUS data were linked to Vektis with probabilistic linkage based on date of birth, sex and postal code, with registrations from 1 January 2011 until 31 December 2017. We defined incident T2D as a registration of one of the considered codes in either one of the databases, and not having T2D at baseline based on self-report, medication use, glucose or HbA1c levels.

### Microbiome

Participants received fecal collection tubes and were asked to bring a fresh fecal sample (within 6 hours after collection) to the study location. In case this was not possible, they were asked to store the sample overnight in a freezer. Samples were stored at −20° at the study visit location for a maximum of 1 day, and transported to the central −80° freezer. Samples of participants who had diarrhoea in the week prior to collection or used antibiotics within 3 months prior to collection were excluded. Samples were shipped to the Wallenberg Laboratory (Sahlgrenska Academy at University of Gothenburg, Sweden) for sequencing. Details of the laboratory procedure and read preprocessing pipeline have been published elsewhere.^19^ In brief, fecal microbiota composition was determined by sequencing the V4 region of the 16S rRNA gene. Both alpha and beta-diversity were used in the statistical analyses. The Shannon index was used to assess alpha diversity. Before calculating beta-diversity, data were centered log-ratio (clr) transformed and rare taxa (present in less than *k%* of samples, where *k = minimal_ read_count×10*^*-3*^) were excluded, in accordance with common practice.^20^ This was done in order to counteract the sparse nature of the data to facilitate downstream analyses. Beta-diversity was calculated using principal component analysis (PCA) with Horn’s method for principal component (PC) selection. Beta-diversity was characterized by six different microbiome PCs (MPCs, online supplemental files 2 and 3). The contributions of operational taxonomical units (OTUs) to the MPCs are shown in online supplemental file 2. In summary, the predominant contributors to MPC1 are from the phylum Firmicutes, specifically within the class Clostridia. Family members include Lachnospiraceae, Christensenellaceae and Ruminococcaceae. Predominant contributors to MPC2 are from the phylum Bacteroidetes and Firmicutes. Family members include Prevotellaceae and Erysipelotrichac. Major contributors to MPC3 are from the phylum Firmicutss, Family members include Ruminococcaceae and Lachnospiraceae. The phylum Bacteroidetes with, for example, Family Bacteroidaceae contributes to MPC4. The phylum Firmicutes contributes to MPC5, for example, with Family Lachnospiraceae and Ruminococcacea. Ruminococcaceau family members including Ruminococcus_1 bicirculans are contributors. Finally, phyla Bacteroidetes, Firmicutes and Actinobacteria are among the ones contributing to MPC6.

### Sphingolipids

Blood plasma sphingolipids were determined in plasma from blood samples collected after at least 10 hours of fasting, using liquid chromatography-tandem mass spectrometry (LC-tMS), further details of which are summarized elsewhere.^8^ All sphingolipids were converted to z-scores. PCA was carried out to reduce sphingolipid data dimensionality^8^ with number of PCs determined using Horn’s parallel method.^21 22^ Based on Horn’s parallel method, two sphingolipid PCs (SPCs) were identified. Plotting the loadings of SPC1 and SPC2 in terms of the component sphingolipids (online supplemental file 4) shows that SPC1 consists of positive loadings of all considered sphingolipids. While SPC2 represents a dominance of ceramides species over the more complex sphingolipids, with a ceramide backbone and additional functional groups (eg, lactosylceramides).

### Covariates

Considered covariates included age, sex, ethnicity, education level, body mass index (BMI), alcohol and tobacco use and physical activity. Ethnicity was defined by the individual’s country of birth combined with the parental countries of birth, Surinamese ethnicity was further subdivided, for example, into South-Asian Surinamese according to self-reported ethnic origin. Education level was included as a categorical variable with four levels: elementary or no schooling (1), lower vocational or lower secondary schooling (2), intermediate vocational or intermediate/higher secondary schooling (3), higher vocational schooling or university (4). BMI was calculated from weight and height assessed during a physical examination in lightly clad barefoot subjects and the reported values are means of duplicate measurements. Information on alcohol and tobacco intake was determined based on questionnaires. Alcohol consumption was classified into three categories based on weekly alcohol consumption estimated from self-reported frequency of alcohol intake combined with self-reported typical alcohol dose. The three categories were low intake (0–4 units/week for men, 0–2 for women), moderate intake (5–14 for men, 3–7 for women) and high intake (>14 for men, >7 for women). Pack years of smoking were calculated by multiplying the number of packs smoked per day (pack size=20 cigarettes) by the number of smoking years, both based on self-report. Physical activity was determined based on Short Questionnaire to Assess Health^23^ with minutes of enhancing physical activity per week multiplied by metabolic equivalent intensity score.

### Data analysis

First, baseline characteristics of the subcohort and cases (including the three in the subcohort) were presented by means and SD for continuous normally distributed variables, by medians and IQRs for continuous not-normally distributed variables and by numbers of observations and percentages for categorical variables.

Second, the association of both gut microbiome and sphingolipids with incident T2D was assessed by Cox proportional hazards (CPH) models.^24^ CPH models were fitted using the pseudolikelihood estimator as described by Lin and Ying^25^ to account for the case-cohort design. The microbiome CPH model included T2D as the outcome, and as exposure alpha and beta-diversity (PCs) simultaneously, after ensuring there was no risk of excessive collinearity, judged by a variance inflation factor <5 for all exposures.^26^ The sphingolipid CPH model used sphingolipid PCs as exposure variables and incident T2D as the outcome.

Third, we assessed the association between gut microbiome and sphingolipids, using Procrustes analysis^27^ with permutation testing (100 000 permutations). Principal coordinates analysis (PCoA) was used for data reduction of both the gut microbiome and the sphingolipid data. For gut microbiome, a PCoA was performed on the Bray-Curtis distance matrix, while for sphingolipids, the Euclidean distance matrix was used. For visualization purposes, individual sphingolipid z-scores were regressed on the first two axes of the microbiome PCoA, and statistical significance of regression coefficients was permutation tested (99 permutations). Additionally, enterotypes of all participants were determined according to Arumugam *et al*^28^ and datapoints were colored by enterotype in the visualization. As a sensitivity analysis, the Procrustes permutation testing was repeated with a PCoA on the Euclidean distance matrix for gut microbiome data.

Finally, we assessed whether sphingolipids mediated the association between the microbiome and incident T2D. Microbiome risk factors that were statistically significantly associated with incident T2D were selected as exposures, while sphingolipid PCs that were statistically significant associated with incident T2D were treated as potential mediators (p<0.05). Mediation was carried out using the difference method^29^ using a pair of models: an uncorrected model equal to the microbiome CPH model and a mediator-corrected model equal to the microbiome CPH model with the candidate mediator added. Mediation was calculated as the difference between the total effect (uncorrected model) and the direct effect (mediator-corrected model). Statistical significance of the observed mediation was determined using permutation testing with 10 000 permutations.

Finally, as an additional exploratory analysis, we repeated survival and mediation analyses with OTUs as microbiome exposures. For survival analysis, OTUs were selected using a two-step procedure, as described by Newcombe *et al*.^30^ For the Bayesian variable selection step, a bayesian factor cut-off *BF> 10* was used, corresponding to strong evidence for selecting a variable.^31^ Additionally, based on *a priori* hypotheses, the bacteroidetes-firmicutes ratio (defined as the ratio of the sum of the abundances all OTUs belonging to the phyla bacteroidetes and firmicutes) and enterotype were included in the second step (CPH model). The bacteroidetes-firmicutes ratio was square-root-transformed to counteract positive skewness. Variables with *p<0.05* were selected for mediation analysis. Mediation analysis using the difference method and permutation testing were performed analogously to the main mediation analysis, with OTUs instead of PCs as exposures.

All analyses were adjusted for potential confounders based on literature, including age, sex, ethnicity, education level (as proxy for socioeconomic status), BMI, alcohol intake, tobacco use and physical activity. BMI may be part of the causal pathway, therefore we have conducted sensitivity analyses without BMI considered as a confounder. As an additional sensitivity analysis, survival and mediation analyses were repeated with different metrics for α-diversity. Shannon diversity was replaced by observed richness and inverse Simpson index. Missing data (n=5 in all variables) were imputed using single imputation with predictive mean matching.^32^,^35^ A statistical significance two-sided threshold of *α=0.05* was employed. Bonferroni correction was used for the mediation analyses with family-wise error rate (*FWER) ≤ 0.05*.

Most analyses were conducted in R V.4.0.3^36^ and Rstudio V.4.0.3.^37^ All software dependencies, including all R packages used, are described in online supplemental file 5.

## Results

Approximately half of the subcohort (48.3%) consisted of women, while 63.8% of the cases were women (table 1). Cases were also more often of South-Asian Surinamese ethnicity (75.0% vs 41.5%) and had a lower educational level than individuals in the subcohort.

**Table 1:** Baseline characteristics of the subcohort and incident T2D cases

	Subcohort (n=176)	Cases (n=36)
Age (years)	52 (42–59)	58 (50–61)
Ethnicity (South-Asian Surinamese)	41.5 (73)	75.0 (27)
Sex (female)	48.3 (85)	63.8 (23)
Education level		
Never been to school or elementary	8.0 (14)	25.0 (9)
Lower vocational or lower secondary	26.7 (47)	33.3 (12)
Intermediate vocational or≥intermediate secondary	21.0 (37)	13.9 (5)
Higher vocational or university	44.3 (78)	27.8 (10)
Alcohol consumption (units/week)		
Low (0–4 for men, 0–2 for women)	57.4 (101)	75.0 (27)
Moderate (5–14 for men, 3–7 for women)	30.7 (54)	16.7 (6)
High (>14 for men, >7 for women)	11.9 (21)	8.3 (3)
SQUASH activity score (min/week)	7755 (5506–11708)	5775 (5063–8423)
Smoking (packyears)	0.3 (0.0–9.1)	0.0 (0.0–10.5)
BMI (kg/m^2^)	24.6 (22.7–27.6)	27.8 (25.6–29.8)

Data are median (IQR) or % (n).

BMI, body mass index; T2D, type 2 diabetes.

The gut microbiome (MPC1, MPC2, MPC3 and Shannon diversity) was statistically significantly associated with incident T2D, for example, HR 1.12 (95% CI 1.03 to 1.21) for MPC1 (table 2A). Sphingolipids, specifically SPC2, reflective of a dominance of ceramides species over the more complex sphingolipids, were also statistically significantly associated with incident T2D (HR 2.12 (95% CI 1.50 to 2.98), p=1.7E-5; table 2B). However, no evidence for an association between SPC1, with positive loadings for all considered sphingolipids, and incident T2D was found (HR 1.13 (95% CI 0.90 to 1.14), p=0.31; table 2B). Next, we found that the gut microbiome was associated with sphingolipids (correlation estimate of *0.089, p=0.02*). A visualization of the association shows that especially the *Ruminococcaceae* enterotype was associated with sphingolipids, in particular, with glucosylceramide (d18:2) and lactosylceramide (d18:2) (online supplemental file 6).

**Table 2:** Association of gut microbiome and sphingolipids with incident T2D

(A) Association of gut microbiome with incident T2D		
Explanatory variable	HR (95% CI)	P value
MPC1	1.12 (1.03 to 1.21)	6.1E-3
MPC2	1.17 (1.08 to 1.28)	2.2E-4
MPC3	1.07 (1.01 to 1.14)	2.8E-2
MPC4	1.05 (0.95 to 1.15)	3.5E-1
MPC5	1.06 (0.98 to 1.16)	1.6E-1
MPC6	1.06 (0.95 to 1.19)	2.8E-1
Α-diversity	0.97 (0.95 to 0.99)	1.6E-3

(B) Association of sphingolipids with incident T2D		
Explanatory variable	HR (95% CI)	P value
SPC1	1.13 (0.90 to 1.14)	3.1E-1
SPC2	2.12 (1.50 to 2.98)	1.7E-5

(A) Results of Cox proportional hazard models with microbiome as exposure and incident T2D as outcome. Analyses were adjusted for age, ethnicity, sex, education level, alcohol consumption, physical activity, smoking and body mass index. Microbiome composition is operationalized into: microbiome principal components (MPCs) and alpha diversity determined by the Shannon index. A case-cohort design is used with 176 participants in the subcohort and 33 additional cases, with Lin-Ying correction. HRs are reported along with 95% CIs and corresponding p values.

(B) Results of Cox proportional hazard models with sphingolipid principal components (SPCs) as exposure and incident T2D as outcome. Analyses were adjusted for age, ethnicity, sex, education level, alcohol consumption, physical activity, smoking and body mass index. A case-cohort design is used with 176 participants in the subcohort and 33 additional cases, with Lin-Ying correction. HRs are reported along with 95% CIs and corresponding p values.

MPC, microbiome principal component; SPC, sphingolipid principal component; T2D, type 2 diabetes.

Since MPC1, MPC2, MPC3 and Shannon diversity were statistically significantly associated with incident T2D, we assessed whether this association was mediated by SPC2 (which was also statistically significant associated with T2D). SPC2 partly mediated the association of MPC2 and Shannon diversity with incident T2D (proportion explained=14.9% and 24.8%, p=0.003 and <0.001, respectively), while it suppressed the association of MPC3 with incident T2D (proportion explained=−21.1%; p=0.006; table 3). Nonetheless, associations between all microbiome variables and incident T2D remained after adjustment for the mediator.

**Table 3:** Mediation of the association between gut microbiome and T2D by sphingolipids

Variable mediated by SPC2	HR_tot_ (95% CI)	p_tot_	HR_dir_ (95% CI)	p_dir_	PE%	p_med_
MPC1	1.12 (1.03 to 1.21)	6.1E-3	1.11 (1.03 to 1.20)	5.5E-3	6.2	9.5E-2
MPC2	1.17 (1.08 to 1.28)	2.2E-4	1.15 (1.06 to 1.24)	5.9E-4	14.9	2.6E-3
MPC3	1.07 (1.01 to 1.14)	2.8E-2	1.09 (1.02 to 1.16)	1.3E-2	−21.1	6.0E-3
A-diversity	0.97 (0.95 to 0.99)	1.6E-3	0.98 (0.96 to 1.00)	1.4E-2	24.8	8E-4

Full mediation analysis results. Explanatory variables included represent variables with p<0.05 in the original Cox proportional hazard models (without mediator). HR estimates, including 95% CIs are included for both the model without mediator (total effect, ‘tot’) and the model with mediator (SPC2) included (direct effect, ‘dir’). The proportion explained (PE), displayed as a percentage, is the fraction of the mediated log-hazard to the total log-hazard and the corresponding p-value is the result of permutation testing. Analyses were adjusted for age, ethnicity, sex, education level, alcohol consumption, physical activity, smoking and body mass index.

HRdir, hazard ratio for the directed effect; HRtot, hazard ratio for the total effect; MPC, microbiome principal component; pdir, p=value for the directed effect; PE%, proportion explained; pmed, p value for the mediation; ptot, p value for the total effect; SPC2, sphingolipid principal component; T2D, type 2 diabetes.

Sixteen individual OTUs were selected using two-step Bayesian variable selection, seven of which showed statistically significant associations with risk of T2D in a confounder-corrected CPH model (online supplemental file 7). Enterotype and *bacteroidetes-firmicutes* ratio did not prove statistically significant risk factors for T2D. Mediation analysis, after Bonferroni correction at *FWER = 0.05*, provides evidence for a mediation of the positive associations between *Butyricimonas paravirosa*, *Alistipes inops* and Zotu304 (unknown species belonging to the genus *Lachnospiraceae*) with incident T2D and a suppression of the negative association of an unknown species belonging to the genus *Oscillibacter* and incident T2D (online supplemental file 8).

Sensitivity analyses excluding BMI did not alter our findings (online supplemental file 9). The sensitivity analyses including different α-diversity metrics were also consistent with the main analyses, however the mediation of the association of MPC2 and MPC3 with T2D by SPC2 is no longer statistically significant, while a larger proportion of the association between α-diversity and T2D is explained by SPC2 (online supplemental file 10).

## Discussion

Our study confirmed in people of Dutch and South-Asian Surinamese ethnicity that both gut microbiome composition and blood plasma sphingolipid profiles are associated with T2D risk and that the association between gut microbiome and incident T2D is partly mediated by sphingolipids.

Observational studies have repeatedly shown associations between the gut microbiome and T2D,^2 3^ and these findings could be replicated in our cohort. The association of SPC2 (higher levels of ceramides and lower levels of more complex sphingolipids) with incident T2D has also been shown in earlier research. Ceramides have been implicated in multiple diseases including T2D,^8 38^ for instance, due to the distortion of signaling pathways involved in glucose metabolism, and triggering apoptosis of pancreatic β-cells. Moreover, a Mendelian randomization study identified Cer(d18:1)/Cer(d20:1) as a risk factor for T2D, suggesting a causal association.^9^

As expected, the microbiome was associated with circulating sphingolipids, and our study indicated that the *Ruminococcaceae* enterotype is primarily involved. This is in line with a study on coronary artery disease, in which alterations were identified in both the gut microbiome (*Roseburia, Klebsiella, Clostridium IV* and *ruminococcaceae*) and metabolites including sphingolipids.^39^ There are several mechanisms through which the gut microbiome may impact circulating sphingolipid levels in humans, including de novo synthetization,^12 13^ modulation of the FXR receptor^15^ and metabolization of sphingolipids from dietary intake.^40^ Whether and to what extent these pathways are indeed the drivers of the observed association is of interest for future research.

Importantly, we identified that sphingolipids are not just a risk factor for T2D and also a mediator of the association between gut microbiome composition and T2D risk. Thus, sphingolipids (mainly the higher levels of ceramides and lower levels of more complex sphingolipids) form a pathway through which the gut microbiome contributes to T2D risk. Unravelling the contribution of individual pathways through which the microbiome impacts sphingolipid levels will, therefore, be highly relevant for the development of interventions and therapeutics to prevent or delay the onset of T2D. Promising approaches include personalized dietary advice, either to modulate the gut microbiome composition or to alter the types of dietary sphingolipid intake^41^; but also pharmaceutics that target enzymes controlling ceramide production, such as SPT and ceramide synthases (eg, CERS6).^42^

### Limitations

Several methodological limitations of this study need to be addressed. First, sample size was limited as data from two different HELIUS substudies were used, for which only a part of the participants overlapped, this has also led to the inclusion of a limited number of cases (n=36). Therefore, we were not able to assess whether effects differed by subgroups, for example, based on ethnicity or sex. However, our study successfully used dimensionality reduction to generate evidence for a complex biological relationship, involving multivariate data in a small sample. This approach increases statistical power by summarizing the complexity of the data into a handful of PCs, thus maximizing signal-to-noise ratio, but most importantly takes into account that the gut microbiome is a complex ecosystem in which the abundances of different species are interdependent. Second, our study lacks dietary information. While this information is available for around 25% of the HELIUS cohort, inclusion hereof would have limited sample size too much, constraining statistical power. Future studies need to confirm our findings with adjustments for dietary intake. Finally, mediation analysis relies on the assumption of causality. Causal relationships of microbiome and sphingolipids with T2D are widely accepted and associations were verified in our data. Yet, we cannot exclude reverse causation, or residual confounding. There is limited evidence of a causal relationship in the existing literature for the association between gut microbiome composition and host sphingolipid profiles^13^ and both exposures are measured at baseline in our data, only supporting an association. However, multiple mechanisms have been described by which microbiota can influence host sphingolipid levels.^12 15 40^ Future studies may, for instance conduct a randomized controlled trial to measure the effect of fecal microbiome transplants on sphingolipid levels.

## Conclusions

Altogether, our study suggests that the association between gut microbiome composition and incident T2D is partly mediated by host plasma sphingolipid levels. Future studies should include larger sample sizes and should include data on dietary intake. They may take our results as a starting point to unravel the exact pathways and involved microbiome species, and to develop interventions to improve circulating sphingolipid ratios.

## Supplementary material

10.1136/bmjdrc-2024-004180 online supplemental file 1

## Data Availability

Data are available upon reasonable request. Data may be obtained from a third party and are not publicly available.
