# Supplementary material for: Plasma sphingolipids mediate the association between gut microbiome composition and type 2 diabetes risk in the HELIUS cohort: a case-cohort study
Source: BMJ Open Diabetes Res Care. 2024 Jul 18;12(4):e004180. doi: 10.1136/bmjdrc-2024-004180 (PMC11261679; doi:10.1136/bmjdrc-2024-004180)
Supplement: online supplemental file 1 [file bmjdrc-12-4-s001.docx]

# Supplement

**Supplementary file 1**


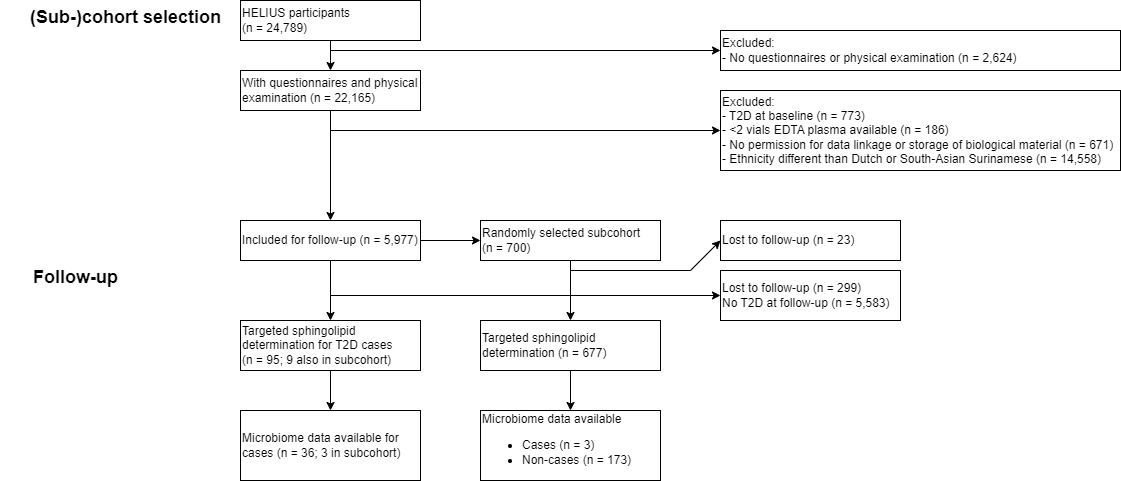
Flow diagram of included participants

**Supplementary file 2**

**Full taxonomy of the crucial contributors to MPC1-6**

| **OTU** | **variable** | **value** | **Kingdom** | **Phylum** | **Class** | **Order** | **Family** | **Genus** | **Species** |
| --- | --- | --- | --- | --- | --- | --- | --- | --- | --- |
| Zotu132 | MPC1 | 0.108682 | Bacteria | Firmicutes | Clostridia | Clostridiales | Lachnospiraceae | Roseburia | NA |
| Zotu64 | MPC1 | 0.121518 | Bacteria | Firmicutes | Clostridia | Clostridiales | Lachnospiraceae | Butyrivibrio | crossotus |
| Zotu103 | MPC1 | 0.106952 | Archaea | Euryarchaeota | Methanobacteria | Methanobacteriales | Methanobacteriaceae | Methanobrevibacter | NA |
| Zotu162 | MPC1 | 0.103422 | Bacteria | Firmicutes | Clostridia | Clostridiales | Christensenellaceae | Christensenellaceae_R-7_group | NA |
| Zotu46 | MPC1 | 0.144459 | Bacteria | Firmicutes | Clostridia | Clostridiales | Christensenellaceae | Christensenellaceae_R-7_group | NA |
| Zotu172 | MPC1 | 0.104327 | Bacteria | Firmicutes | Clostridia | Clostridiales | Ruminococcaceae | NA | NA |
| Zotu84 | MPC1 | 0.112315 | Bacteria | Firmicutes | Clostridia | Clostridiales | Ruminococcaceae | Ruminiclostridium_6 | NA |
| Zotu89 | MPC1 | 0.11225 | Bacteria | Firmicutes | Clostridia | Clostridiales | Ruminococcaceae | Ruminococcaceae_NK4A214_group | NA |
| Zotu39 | MPC1 | 0.109648 | Bacteria | Firmicutes | Clostridia | Clostridiales | Ruminococcaceae | Ruminococcaceae_UCG-002 | bacterium |
| Zotu115 | MPC1 | 0.120082 | Bacteria | Firmicutes | Clostridia | Clostridiales | Ruminococcaceae | Ruminococcaceae_UCG-002 | NA |
| Zotu35 | MPC1 | 0.142136 | Bacteria | Firmicutes | Clostridia | Clostridiales | Ruminococcaceae | Ruminococcaceae_UCG-002 | NA |
| Zotu105 | MPC1 | 0.135888 | Bacteria | Firmicutes | Clostridia | Clostridiales | Ruminococcaceae | Ruminococcaceae_UCG-005 | NA |
| Zotu63 | MPC1 | 0.141117 | Bacteria | Firmicutes | Clostridia | Clostridiales | Ruminococcaceae | Ruminococcaceae_UCG-005 | NA |
| Zotu164 | MPC1 | 0.113516 | Bacteria | Firmicutes | Clostridia | Clostridiales | Ruminococcaceae | Ruminococcaceae_UCG-005 | NA |
| Zotu104 | MPC1 | -0.11144 | Bacteria | Firmicutes | Clostridia | Clostridiales | Lachnospiraceae | NA | NA |
| Zotu26 | MPC2 | 0.105725 | Bacteria | Bacteroidetes | Bacteroidia | Bacteroidales | Bacteroidaceae | Bacteroides | uniformis |
| Zotu61 | MPC2 | 0.163393 | Bacteria | Bacteroidetes | Bacteroidia | Bacteroidales | Rikenellaceae | Alistipes | putredinis |
| Zotu113 | MPC2 | 0.128364 | Bacteria | Bacteroidetes | Bacteroidia | Bacteroidales | Rikenellaceae | Alistipes | NA |
| Zotu1 | MPC2 | -0.23904 | Bacteria | Bacteroidetes | Bacteroidia | Bacteroidales | Prevotellaceae | Prevotella_9 | copri |
| Zotu5 | MPC2 | -0.19789 | Bacteria | Bacteroidetes | Bacteroidia | Bacteroidales | Prevotellaceae | Prevotella_9 | NA |
| Zotu20 | MPC2 | -0.16276 | Bacteria | Bacteroidetes | Bacteroidia | Bacteroidales | Prevotellaceae | Prevotella_9 | NA |
| Zotu29 | MPC2 | 0.11476 | Bacteria | Verrucomicrobia | Verrucomicrobiae | Verrucomicrobiales | Akkermansiaceae | Akkermansia | muciniphila |
| Zotu31 | MPC2 | -0.13307 | Bacteria | Firmicutes | Negativicutes | Selenomonadales | Veillonellaceae | Dialister | NA |
| Zotu43 | MPC2 | -0.1218 | Bacteria | Firmicutes | Negativicutes | Selenomonadales | Veillonellaceae | Megasphaera | NA |
| Zotu52 | MPC2 | -0.12438 | Bacteria | Firmicutes | Negativicutes | Selenomonadales | Acidaminococcaceae | Phascolarctobacterium | succinatutens |
| Zotu50 | MPC2 | 0.105298 | Bacteria | Firmicutes | Erysipelotrichia | Erysipelotrichales | Erysipelotrichaceae | Erysipelotrichaceae_UCG-003 | NA |
| Zotu25 | MPC2 | -0.17154 | Bacteria | Firmicutes | Erysipelotrichia | Erysipelotrichales | Erysipelotrichaceae | Catenibacterium | mitsuokai |
| Zotu34 | MPC2 | -0.13544 | Bacteria | Firmicutes | Erysipelotrichia | Erysipelotrichales | Erysipelotrichaceae | Holdemanella | NA |
| Zotu27 | MPC2 | 0.119058 | Bacteria | Firmicutes | Clostridia | Clostridiales | Ruminococcaceae | Ruminococcus_1 | bicirculans |
| Zotu103 | MPC3 | 0.129225 | Archaea | Euryarchaeota | Methanobacteria | Methanobacteriales | Methanobacteriaceae | Methanobrevibacter | NA |
| Zotu140 | MPC3 | 0.121444 | Bacteria | Firmicutes | Clostridia | Clostridiales | NA | NA | NA |
| Zotu92 | MPC3 | 0.138857 | Bacteria | Firmicutes | Clostridia | Clostridiales | Christensenellaceae | NA | NA |
| Zotu14 | MPC3 | -0.10582 | Bacteria | Firmicutes | Clostridia | Clostridiales | Ruminococcaceae | Faecalibacterium | CM04-06 |
| Zotu44 | MPC3 | -0.10013 | Bacteria | Firmicutes | Clostridia | Clostridiales | Ruminococcaceae | Faecalibacterium | NA |
| Zotu84 | MPC3 | 0.100913 | Bacteria | Firmicutes | Clostridia | Clostridiales | Ruminococcaceae | Ruminiclostridium_6 | NA |
| Zotu90 | MPC3 | -0.12645 | Bacteria | Firmicutes | Clostridia | Clostridiales | Lachnospiraceae | CAG-56 | NA |
| Zotu82 | MPC3 | -0.1002 | Bacteria | Firmicutes | Clostridia | Clostridiales | Lachnospiraceae | NA | NA |
| Zotu55 | MPC4 | 0.155229 | Bacteria | Bacteroidetes | Bacteroidia | Bacteroidales | Bacteroidaceae | Bacteroides | NA |
| Zotu78 | MPC4 | 0.106681 | Bacteria | Bacteroidetes | Bacteroidia | Bacteroidales | Bacteroidaceae | Bacteroides | caccae |
| Zotu61 | MPC4 | 0.128328 | Bacteria | Bacteroidetes | Bacteroidia | Bacteroidales | Rikenellaceae | Alistipes | putredinis |
| Zotu1 | MPC4 | 0.110731 | Bacteria | Bacteroidetes | Bacteroidia | Bacteroidales | Prevotellaceae | Prevotella_9 | copri |
| Zotu70 | MPC4 | 0.164215 | Bacteria | Bacteroidetes | Bacteroidia | Bacteroidales | Bacteroidaceae | Bacteroides | massiliensis |
| Zotu73 | MPC4 | 0.163312 | Bacteria | Bacteroidetes | Bacteroidia | Bacteroidales | Tannerellaceae | Parabacteroides | merdae |
| Zotu64 | MPC4 | -0.10295 | Bacteria | Firmicutes | Clostridia | Clostridiales | Lachnospiraceae | Butyrivibrio | crossotus |
| Zotu69 | MPC4 | -0.10534 | Bacteria | Firmicutes | Clostridia | Clostridiales | Peptostreptococcaceae | Intestinibacter | bartlettii |
| Zotu120 | MPC4 | 0.107076 | Bacteria | Proteobacteria | Gammaproteobacteria | Betaproteobacteriales | Burkholderiaceae | Sutterella | NA |
| Zotu8 | MPC4 | 0.113513 | Bacteria | Actinobacteria | Actinobacteria | Bifidobacteriales | Bifidobacteriaceae | Bifidobacterium | NA |
| Zotu15 | MPC4 | 0.114316 | Bacteria | Actinobacteria | Coriobacteriia | Coriobacteriales | Coriobacteriaceae | Collinsella | aerofaciens |
| Zotu58 | MPC4 | 0.11678 | Bacteria | Firmicutes | Negativicutes | Selenomonadales | Veillonellaceae | Megamonas | funiformis |
| Zotu87 | MPC4 | -0.10317 | Bacteria | Firmicutes | Clostridia | Clostridiales | Clostridiaceae_1 | Clostridium_sensu_stricto_1 | NA |
| Zotu65 | MPC5 | -0.11944 | Bacteria | Bacteroidetes | Bacteroidia | Bacteroidales | Bacteroidaceae | Bacteroides | NA |
| Zotu17 | MPC5 | -0.10194 | Bacteria | Bacteroidetes | Bacteroidia | Bacteroidales | Bacteroidaceae | Bacteroides | NA |
| Zotu16 | MPC5 | 0.100098 | Bacteria | Firmicutes | Clostridia | Clostridiales | Lachnospiraceae | Agathobacter | NA |
| Zotu64 | MPC5 | -0.13229 | Bacteria | Firmicutes | Clostridia | Clostridiales | Lachnospiraceae | Butyrivibrio | crossotus |
| Zotu81 | MPC5 | -0.10276 | Bacteria | Firmicutes | Clostridia | Clostridiales | Lachnospiraceae | Anaerostipes | hadrus |
| Zotu209 | MPC5 | 0.123541 | Bacteria | Firmicutes | Clostridia | Clostridiales | Peptostreptococcaceae | Terrisporobacter | NA |
| Zotu69 | MPC5 | 0.146297 | Bacteria | Firmicutes | Clostridia | Clostridiales | Peptostreptococcaceae | Intestinibacter | bartlettii |
| Zotu120 | MPC5 | -0.11255 | Bacteria | Proteobacteria | Gammaproteobacteria | Betaproteobacteriales | Burkholderiaceae | Sutterella | NA |
| Zotu118 | MPC5 | -0.14223 | Bacteria | Proteobacteria | Gammaproteobacteria | Betaproteobacteriales | Burkholderiaceae | Sutterella | wadsworthensis |
| Zotu45 | MPC5 | 0.113298 | Bacteria | Firmicutes | Negativicutes | Selenomonadales | Veillonellaceae | Dialister | invisus |
| Zotu25 | MPC5 | 0.113023 | Bacteria | Firmicutes | Erysipelotrichia | Erysipelotrichales | Erysipelotrichaceae | Catenibacterium | mitsuokai |
| Zotu24 | MPC5 | 0.121232 | Bacteria | Firmicutes | Bacilli | Lactobacillales | Streptococcaceae | Streptococcus | NA |
| Zotu27 | MPC5 | 0.161298 | Bacteria | Firmicutes | Clostridia | Clostridiales | Ruminococcaceae | Ruminococcus_1 | bicirculans |
| Zotu106 | MPC5 | 0.101246 | Bacteria | Firmicutes | Clostridia | Clostridiales | Ruminococcaceae | Ruminococcus_1 | callidus |
| Zotu432 | MPC5 | 0.107098 | Bacteria | Firmicutes | Clostridia | Clostridiales | Ruminococcaceae | Ruminiclostridium_5 | NA |
| Zotu262 | MPC5 | 0.125868 | Bacteria | Firmicutes | Clostridia | Clostridiales | Ruminococcaceae | Ruminococcaceae_UCG-004 | NA |
| Zotu60 | MPC5 | -0.10196 | Bacteria | Firmicutes | Clostridia | Clostridiales | Lachnospiraceae | Lachnoclostridium | NA |
| Zotu78 | MPC6 | -0.16193 | Bacteria | Bacteroidetes | Bacteroidia | Bacteroidales | Bacteroidaceae | Bacteroides | caccae |
| Zotu91 | MPC6 | 0.128779 | Bacteria | Bacteroidetes | Bacteroidia | Bacteroidales | Prevotellaceae | Prevotella_7 | NA |
| Zotu1 | MPC6 | -0.16569 | Bacteria | Bacteroidetes | Bacteroidia | Bacteroidales | Prevotellaceae | Prevotella_9 | copri |
| Zotu20 | MPC6 | -0.18571 | Bacteria | Bacteroidetes | Bacteroidia | Bacteroidales | Prevotellaceae | Prevotella_9 | NA |
| Zotu116 | MPC6 | 0.144681 | Bacteria | Bacteroidetes | Bacteroidia | Bacteroidales | Prevotellaceae | Alloprevotella | NA |
| Zotu28 | MPC6 | 0.166753 | Bacteria | Firmicutes | Clostridia | Clostridiales | Lachnospiraceae | Roseburia | intestinalis |
| Zotu181 | MPC6 | 0.119615 | Bacteria | Proteobacteria | Deltaproteobacteria | Desulfovibrionales | Desulfovibrionaceae | Desulfovibrio | NA |
| Zotu8 | MPC6 | -0.11507 | Bacteria | Actinobacteria | Actinobacteria | Bifidobacteriales | Bifidobacteriaceae | Bifidobacterium | NA |
| Zotu23 | MPC6 | -0.12679 | Bacteria | Actinobacteria | Actinobacteria | Bifidobacteriales | Bifidobacteriaceae | Bifidobacterium | NA |
| Zotu151 | MPC6 | -0.13068 | Bacteria | Actinobacteria | Actinobacteria | Bifidobacteriales | Bifidobacteriaceae | Bifidobacterium | bifidum |
| Zotu110 | MPC6 | -0.10684 | Bacteria | Actinobacteria | Actinobacteria | Bifidobacteriales | Bifidobacteriaceae | Bifidobacterium | NA |
| Zotu149 | MPC6 | 0.1028 | Bacteria | Actinobacteria | Coriobacteriia | Coriobacteriales | Eggerthellaceae | Enterorhabdus | NA |
| Zotu52 | MPC6 | 0.183577 | Bacteria | Firmicutes | Negativicutes | Selenomonadales | Acidaminococcaceae | Phascolarctobacterium | succinatutens |
| Zotu107 | MPC6 | -0.12927 | Bacteria | Firmicutes | Negativicutes | Selenomonadales | Acidaminococcaceae | Phascolarctobacterium | faecium |
| Zotu25 | MPC6 | 0.116473 | Bacteria | Firmicutes | Erysipelotrichia | Erysipelotrichales | Erysipelotrichaceae | Catenibacterium | mitsuokai |
| Zotu241 | MPC6 | -0.10811 | Bacteria | Firmicutes | Erysipelotrichia | Erysipelotrichales | Erysipelotrichaceae | Turicibacter | sanguinis |
| Zotu97 | MPC6 | -0.10086 | Bacteria | Firmicutes | Clostridia | Clostridiales | Clostridiaceae_1 | Clostridium_sensu_stricto_1 | NA |
| Zotu59 | MPC6 | 0.111955 | Bacteria | Firmicutes | Clostridia | Clostridiales | Ruminococcaceae | NA | NA |
| Zotu27 | MPC6 | 0.125146 | Bacteria | Firmicutes | Clostridia | Clostridiales | Ruminococcaceae | Ruminococcus_1 | bicirculans |
| Zotu9 | MPC6 | 0.21174 | Bacteria | Firmicutes | Clostridia | Clostridiales | Ruminococcaceae | Ruminococcus_2 | bromii |

Columns - from the left - contain: OTU (Operational Taxonomic Unit) name, MPC (Microbiome Principal Component) number, loading value, taxonomic classification. Only species with |loading|$>0.1$ are shown, which corresponds to the red horizontal cut-off lines in supplementary file 4.

**Supplementary file 3**

A summary of the loadings of microbiome principal components (MPC1-6), microbiome variables that were associated with incident T2D. Individual operational taxonomical units (OTUs) are coloured by phylum and only the four phyla with the highest number of OTUs are shown. Red lines at y = −0.1 and y = 0.1 are an arbitrary cutoff to indicate OTUs with high contribution to MPCs. OTUs with loadings exceeding these cut-off values are summarized in supplementary file 2.


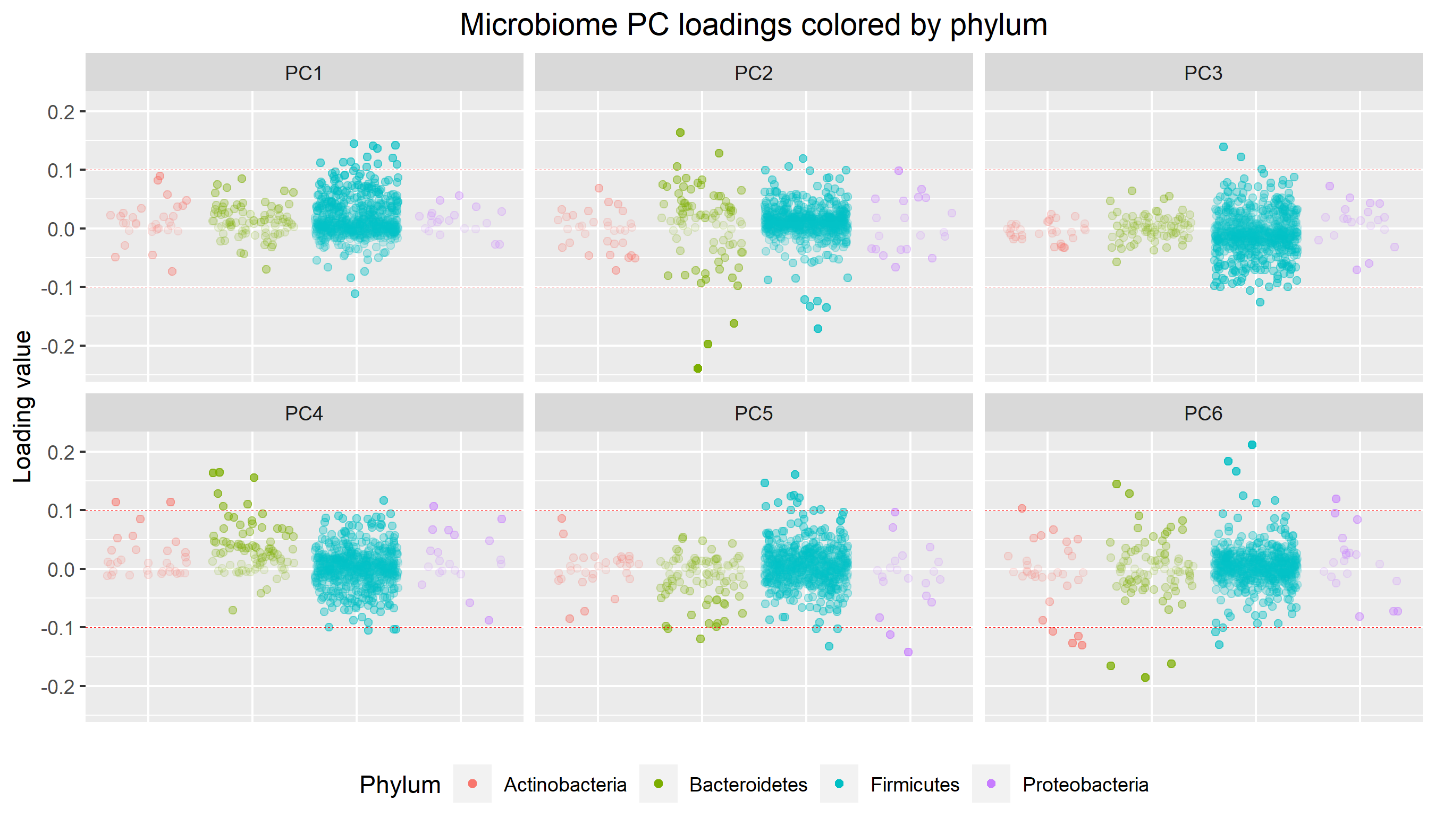


**Supplementary file 4**

Bar plots of sphingolipid principal component (PC) loadings. The X-axis represents loadings, with positive values synonymous with a positive contribution to the PC and greater absolute values meaning a more important role in determining PC value. Bars are coloured by metabolite type with (no additional functional groups) ceramides shown in red and more complex sphingolipid species shown in blue.


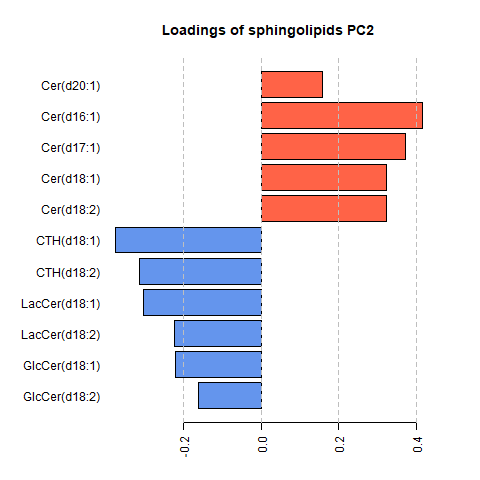

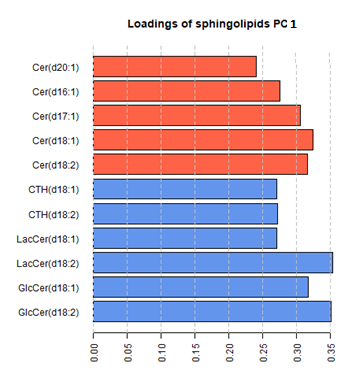


## Supplementary file 5

Predictive mean matching (PMM) was carried out using the function mice from the package mice (v3.14.0)(44). Centered log-ratio (clr) transformation was carried out with the transform function of the microbiome (v1.17.41) package(45). Taxa pruning was implemented using the prune_taxa function of the phyloseq (v1.34.0) package(46). Horn’s parallel method was carried out using the function paran from package paran (v1.5.2)(47). Observed richness and Shannon diversity were calculated using the estimate_richness function of the phyloseq (v1.34.0) package. Bray-Curtis distance was calculated using vegdist from the vegan (v2.5.7) package(48). PCoA implementation from the ape (v5.6.2) package(49) was used. Procrustes analysis was carried out using the procrustes function from vegan (v2.5.7). Regression of sphingolipids on microbiome PCoA together with permutation testing were performed using the envfit function from vegan (v2.5.7) and plotting was performed using ggvegan (v0.1.0) functionality(50). Survival analysis was implemented using the cch function from the survival (v3.3.1) package(51). Functional fit of martingale residuals was plotted with the help of the lowess function from the stats (v4.0.3) package(33). Mediation analysis was implemented manually. BVS was performed using the Java (v8.0) BGLiMS package, via the R interface R2BGLiMS (v0.1.7.2.2020)(52). Remaining dependencies consist of the following R packages: tidyverse(v1.3.1) (53), haven (v2.4.3), reshape2 (v1.4.4)(54), moments (v0.14)(55), IMIFA (v2.1.8)(56), compositions (v2.0.4)(57), PERFect (v0.2.4)(58), mitml (v0.4.3)(59), ppcor (v1.1)(60) and wesanderson (v0.3.6)(61).

**Supplementary file 6**

Plot of participant gut microbiome profiles against the first two axes of the microbiome Principal Coordinates Analysis (PCoA). Points are colored by enterotype, according to Arumugam et al. (2011). Arrows represent the vectors composed of regression coefficients of sphingolipid values on both PCoA axes and are only shown if p < 0.05 in permutation testing. Several sphingolipids are associated with the gut microbiome. All included sphingolipids show negative regression coefficients with PCoA axis 2, suggesting that changes along this axis are associated with decreases in all sphingolipids shown in the figure.


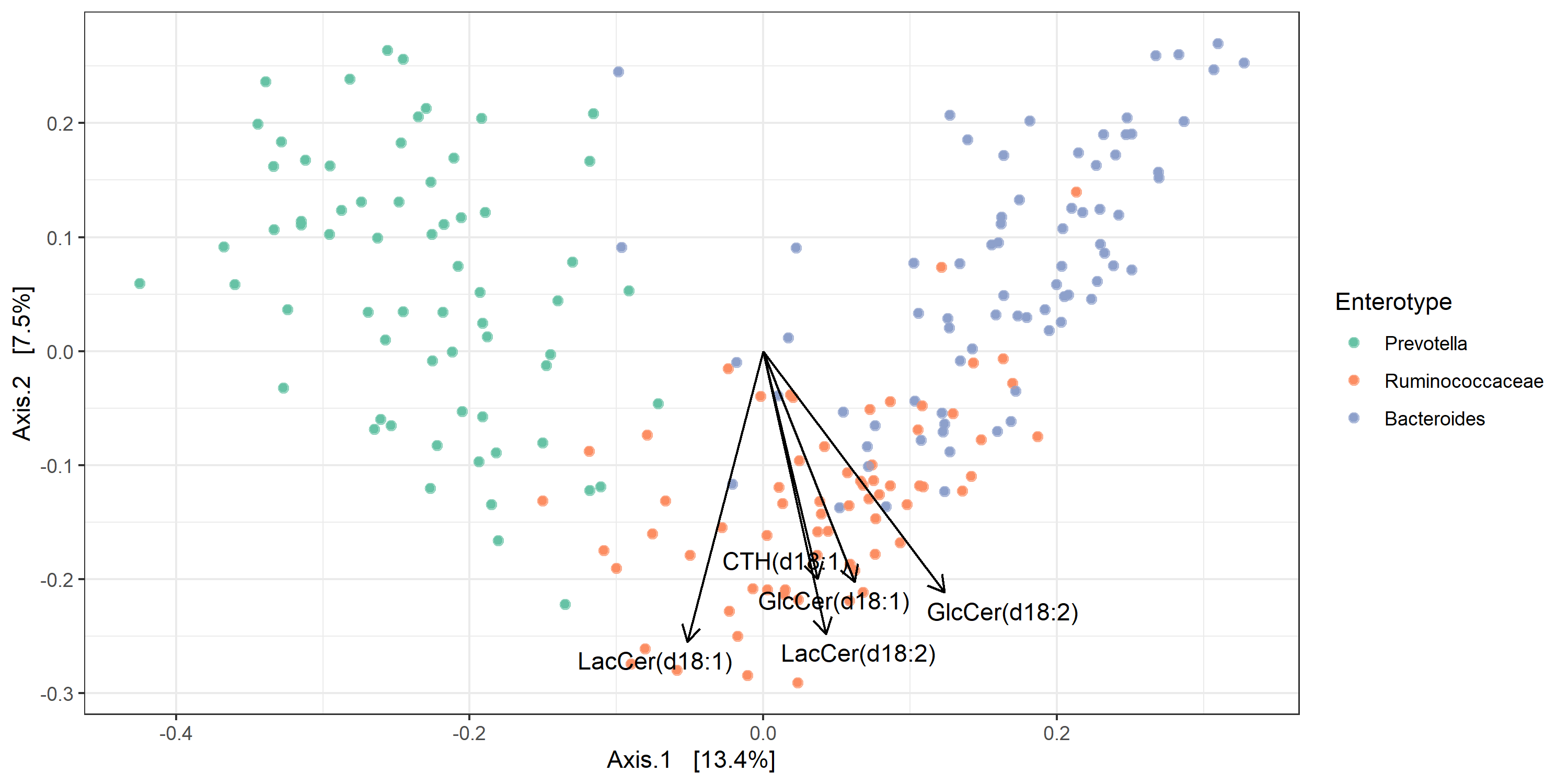


**Supplementary file 7.** Individual OTUs associated with T2D.

| **Expl var** | **BF** | **HR (95CI)** | **p-value** |
| --- | --- | --- | --- |
| Zotu566 (*Butyricimonas paravirosa*) | 13.7 | 2.86 (1.70-4.82) | 7.3E-5 |
| Zotu218 (*Alistipes inops*) | 10.9 | 2.03 (1.39-2.98) | 2.6E-4 |
| Zotu551 (*Ruminococcaceae sp.*) | 10.9 | 0.71 (0.31-1.67) | 4.4E-1 |
| Zotu371 (unknown sp.) | 34.4 | 0.41 (0.21-0.80) | 9.3E-3 |
| Zotu304 (*Lachnospiraceae sp.*) | 13.3 | 2.02 (1.23-3.32) | 5.5E-3 |
| Zotu319 (unknown sp.) | 10.9 | 0.71 (0.37-1.34) | 2.9E-1 |
| Zotu87 (*Clostridium sp.*) | 29.8 | 0.94 (0.68-1.29) | 6.9E-1 |
| Zotu13641 (*Faecalibacterium sp.*) | 16.7 | 0.39 (0.13-1.14) | 8.5E-2 |
| Zotu21939 (*Subdoligranulum sp.*) | 10.1 | 0.029 (0.002-0.485) | 1.4E-2 |
| Zotu1212 (*Phocea massiliensis*) | 14.5 | 1.95 (0.88-4.33) | 1.0E-1 |
| Zotu139 (*Butyricicoccus sp.*) | 14.3 | 0.77 (0.57-1.04) | 9.2E-2 |
| Zotu646 (*Ruminococcaceae sp.*) | 10.3 | 0.43 (0.12-1.55) | 2.0E-1 |
| Zotu263 (*Ruminococcaceae sp.*) | 11.7 | 0.68 (0.38-1.24) | 2.1E-1 |
| Zotu608 (*Oscillibacter sp.*) | 54.9 | 0.30 (0.12-0.71) | 6.3E-3 |
| Zotu262 (*Ruminococcaceae sp.*) | 11.3 | 1.40 (1.06-1.85) | 2.0E-3 |
| Zotu320 (*Marvinbryantia sp.*) | 11.9 | 0.62 (0.36-1.07) | 8.3E-2 |
| Ruminococcaceae enterotype | - | 0.74 (0.08-7.02) | 8.0E-1 |
| Bacteroides enterotype | - | 0.31 (0.04-2.17) | 2.4E-1 |
| $\sqrt{bacteroides/firmicutes}$ | - | 1.97 (0.10-38.39) | 6.5E-1 |

**Supplementary file 8.** Mediation of the association of individual OTUs with T2D by sphingolipids.

| **Variable mediated by SPC2** |  | **HR_tot_ (95CI)** | ***p*_tot_** | **HR_dir_ (95CI)** | ***p*_dir_** | **PE%** | ***p*_med_** |
| --- | --- | --- | --- | --- | --- | --- | --- |
| Zotu566 (*Butyricimonas paravirosa*) |  | 2.74 (1.69-4.43) | 7.3E-5 | 2.33 (1.42-3.82) | 7.8E-4 | 16.0 | 4.8E-3 |
| Zotu218 (*Alistipes inops*) |  | 1.92 (1.34-2.74) | 2.6E-4 | 1.40 (0.98-2.01) | 6.6E-2 | 47.9 | 1.2E-3 |
| Zotu371 (unknown sp.) |  | 0.48 (0.27-0.85) | 9.3E-3 | 0.68 (0.39-1.17) | 1.6E-2 | 23.7 | 6.7E-2 |
| Zotu304 (*Lachnospiraceae sp.*) |  | 2.12 (1.31-3.45) | 5.5E-3 | 1.65 (1.00-2.73) | 5.1E-2 | 47.3 | $<$2.0E-4 |
| Zotu21939 (*Subdoligranulum sp.*) |  | 0.028 (0.002-0.432) | 1.4E-2 | 0.048 (0.004-0.591) | 1.8E-2 | 33.4 | 1.2E-2 |
| Zotu608 (*Oscillibacter sp.*) |  | 0.32 (0.15-0.68) | 6.3E-3 | 0.29 (0.13-0.64) | 2.0E-3 | -50.1 | 3E-3 |
| Zotu262 (*Ruminococcaceae sp.*) |  | 1.41 (1.07-1.86) | 2.0E-3 | 1.25 (0.95-1.65) | 1.1E-1 | -92.0 | 1.4E-1 |

**Supplementary file 9. Sensitivity analysis excluding BMI as covariate. Mediation of the association between gut microbiome and T2D by sphingolipids.**

| **Variable mediated by SPC2** | **HR_tot_ (95%-CI)** | ***p*_tot_** | **HR_dir_ (95%-CI)** | ***p*_dir_** | **PE%** | ***p*_med_** |
| --- | --- | --- | --- | --- | --- | --- |
| **MPC1** | 1.08 (0.99 - 1.17) | 7.5E-2 | 1.09 (1.02 - 1.18) | 1.6E-2 | -22.6 | 6.4E-3 |
| **MPC2** | 1.13 (1.04 - 1.23) | 5.0E-3 | 1.13 (1.04 - 1.21) | 1.9E-3 | 4.3 | 1.4E-1 |
| **MPC3** | 1.06 (1.00 - 1.13) | 4.4E-2 | 1.09 (1.02 - 1.17) | 1.2E-2 | -40.3 | 2.0E-4 |
| **Shannon diversity** | 0.98 (0.96 – 1.00) | 2.2E-2 | 0.98 (0.96 - 1.00) | 3.4E-2 | 15.5 | 7.4E-3 |

**Supplementary file 10. Sensitivity analysis using Observed richness and Inverse Simpson index as alpha-diversity metrics. Mediation of the association between gut microbiome and T2D by sphingolipids.**

| **Variable mediated by SPC2** | **HR_tot_ (95%-CI)** | ***p*_tot_** | **HR_dir_ (95%-CI)** | ***p*_dir_** | **PE%** | ***p*_med_** |
| --- | --- | --- | --- | --- | --- | --- |
| **MPC1** | 1.06 (0.99 - 1.15) | 1.0E-1 | 1.09 (1.01 - 1.17) | 2.7E-2 | -29.1 | 4.6E-3 |
| **MPC2** | 1.13 (1.04 - 1.23) | 3.1E-3 | 1.12 (1.04 - 1.21) | 3.4E-3 | 8.5 | 7.5E-2 |
| **MPC3** | 1.08 (1.01 - 1.15) | 1.7E-2 | 1.09 (1.02 - 1.17) | 1.6E-2 | -12.0 | 6.6E-2 |
| **Inverse Simpson index** | 0.93 (0.88 – 0.99) | 1.9E-2 | 0.96 (0.90 - 1.01) | 1.2E-1 | 34.1 | 5.2E-3 |

*Observed richness was excluded from the mediation analyses, since no association was observed with T2D. HR_tot_ 1.00 95%-CI 1.00 – 1.01 (p-value 0.98).*
